# The Nitric Oxide Pathway Provides Innate Antiviral Protection in Conjunction with the Type I Interferon Pathway in Fibroblasts

**DOI:** 10.1371/journal.pone.0031688

**Published:** 2012-02-21

**Authors:** Devangi R. Mehta, Ali A. Ashkar, Karen L. Mossman

**Affiliations:** Department of Pathology and Molecular Medicine, McMaster Immunology Research Centre, Michael DeGroote Institute for Infectious Disease Research, McMaster University, Hamilton, Ontario, Canada; Southern Illinois University School of Medicine, United States of America

## Abstract

The innate host response to virus infection is largely dominated by the production of type I interferon and interferon stimulated genes. In particular, fibroblasts respond robustly to viral infection and to recognition of viral signatures such as dsRNA with the rapid production of type I interferon; subsequently, fibroblasts are a key cell type in antiviral protection. We recently found, however, that primary fibroblasts deficient for the production of interferon, interferon stimulated genes, and other cytokines and chemokines mount a robust antiviral response against both DNA and RNA viruses following stimulation with dsRNA. Nitric oxide is a chemical compound with pleiotropic functions; its production by phagocytes in response to interferon-γ is associated with antimicrobial activity. Here we show that in response to dsRNA, nitric oxide is rapidly produced in primary fibroblasts. In the presence of an intact interferon system, nitric oxide plays a minor but significant role in antiviral protection. However, in the absence of an interferon system, nitric oxide is critical for the protection against DNA viruses. In primary fibroblasts, NF-κB and interferon regulatory factor 1 participate in the induction of inducible nitric oxide synthase expression, which subsequently produces nitric oxide. As large DNA viruses encode multiple and diverse immune modulators to disable the interferon system, it appears that the nitric oxide pathway serves as a secondary strategy to protect the host against viral infection in key cell types, such as fibroblasts, that largely rely on the type I interferon system for antiviral protection.

## Introduction

A central theme today in medical research is understanding the delicate balance of interactions between a pathogen and its host. These interactions dictate the pathological consequences of infections. It is well recognized that the innate immune response against pathogens focuses on detection of highly conserved pathogen-associated molecular patterns (PAMPs) that are distinct from the host. Host pathogen recognition receptors have evolved to recognize patterns such as nucleic acids from pathogens including bacteria and viruses [1]. The first line of defence against invading pathogens is the rapid and robust production of type I interferon (IFN), a family of cytokines with potent immune stimulatory and pathogen-controlling properties. Fibroblasts are amongst the first cell types involved in the line of defence against numerous pathogens. Fibroblasts are widely distributed in organisms [2] and play an important role in the transition from innate to adaptive immunity [3], [4]. This role is largely a result of cytokine production [5], most notably IFNβ, which was originally termed fibroblast IFN. As such, fibroblasts are important effectors of the early innate immune response. Indeed, in a recent study, non-hematopoietic cells, and fibroblasts in particular, were shown to mediate protection against an emerging viral infection through a type I IFN response [6].

As all viruses are thought to make double-stranded RNA (dsRNA) as a by-product of their replication cycles, this molecule is a potent producer of type I IFN and as such, is commonly used to study innate immune responses to virus infection. DsRNA can be recognized by three different families of pathogen recognition receptors, the toll-like receptors (TLRs), the retinoic acid inducible gene I (RIG-I)-like receptors (RLRs) and the nucleotide oligomerization domain-like receptors (NLRs); these bind dsRNA and initiate cellular signaling pathways [7], [8]. The TLRs and RLRs elicit antiviral pathways involving type I and type III IFNs and cytokine production, whereas NLRs elicit caspase 1 activation for IL-1β maturation [7], [8]. In fibroblasts, IFN is typically made upon detection of viral dsRNA by TLR-3 and the RLRs, RIG-I and melanoma differentiation-associated gene 5 (MDA5). These pathways converge on NF-κB and IFN regulatory factor 3 (IRF3), which, upon activation, are important for cytokine and IFNβ production. Type I IFN, such as IFNβ, signal through the JAK-STAT pathway, which includes IRF9 as an essential component. This signaling leads to the induction of IRF7, which amplifies the cellular antiviral response through the generation of IFNα species and IFN stimulated genes (ISGs) [8].

In the prototypic antiviral response, IRF3 and early production of type I IFN and ISGs are arguably the most important events in combating infection. In humans, inborn errors that impair the production of, or responsiveness to, either of the three classes of IFN increase susceptibility to mycobacterial and viral infection [9]. Viral infection of mice deficient for IFNβ or type I IFN signaling is typically a lethal event, even under low multiplicity infection conditions [10]–[13]. Similarly, in the absence of IRF3, mice are more susceptible to virus infection due to a 20–50 fold reduction in type I IFN expression [14]. However, infection of mouse embryonic fibroblasts (MEFs) deficient for IRF3 with Newcastle disease virus induced a number of IRF3-independent direct response genes, including several p200 family proteins [15]. We have also observed IFN and ISG induction and a subsequent antiviral response to long dsRNA molecules independent of IRF3 [16]. Alternatively, ISG induction and antiviral protection can be independent of IFN due to IRF3 binding directly to the promoter of a subset of ISGs [17]. Despite the presence of multiple pathways, the type I IFN system dominates the antiviral response in non-hematopoietic cells such as fibroblasts.

To ensure their success, however, viruses such as the highly successful human herpes simplex virus (HSV)-1 inactivate IRF3 [18]–[21] and subvert the type I IFN response [22]. In fact, over 200 anti-IRF3 and anti-IFN mechanisms encoded by diverse viruses have been identified [23]–[25]. In general, DNA viruses are particularly adept at subverting the type I IFN system, particularly the large DNA viruses that encode multiple immune response modifiers. Given the importance of IRF3 and type I IFN in protection against virus infection, it is likely that all viruses encode mechanisms to disable these proteins. Accordingly, there are compensatory mechanisms to protect the host in the event that either of these crucial proteins is compromised. While there have been numerous studies examining either the IRF3-independent or the IFN-independent antiviral response, until recently, it was unknown if the host could be protected if both IRF3 and IFN were absent.

We have previously observed a protective response against both DNA and RNA viruses in the absence of IRF3 and IRF9 in primary MEFs [16]. In response to dsRNA, MEFs deficient for IRF3 and IRF9 fail to induce other IRFs, IFN or ISGs, suggesting that the antiviral response observed in these cells is independent of the type I IFN system [16]. In the absence of IFN and ISGs, there is approximately 60–90% inhibition of vesicular stomatitis virus (VSV) and HSV-1 replication following poly I∶C stimulation. Interestingly, the earlier and more potent response occurs against HSV-1 [16], which is well known for its ability to subvert the host response in comparison to VSV, which is highly susceptible to the effects of type I IFN [26]–[28].

Given the importance of the type I IFN system as the primary antiviral defence mechanism in non-hematopoietic cells, it is unknown how these cells protect themselves in the absence of this system. There are other innate molecules induced by viral infection that play a role in the host antiviral response. One such molecule is nitric oxide. Endogenously produced nitric oxide has many biological functions including smooth muscle relaxation and neurotransmission [29]. This molecule also plays an important antimicrobial role against numerous pathogens [30]–[32]. Although the antibacterial effects of nitric oxide are well appreciated, nitric oxide is also effective in the clearance of viruses, particularly DNA viruses [33]–[35]; these effects can be independent of IFN and ISGs. Nitric oxide is synthesized by nitric oxide synthase (NOS) of which there are three known isoforms, endothelial (e), neuronal (n) and inducible (i) [29]. In the context of protection against invading pathogens, the inducible nitric oxide (iNOS) is mainly associated with phagocytes and other immune cells following induction of tumor necrosis factor-alpha and IFN-γ. Here, we have investigated the potential role played by nitric oxide in the antiviral response observed against HSV-1 in MEFs in the absence of IFN production and signaling. We found that in MEFs, nitric oxide is made by iNOS in response to dsRNA and plays an important role in the antiviral state observed in the absence of IRF3 and IFN production.

## Results

### Poly I∶C induces production of nitric oxide in the absence of IRF3 and IRF9

**Figure 1:**
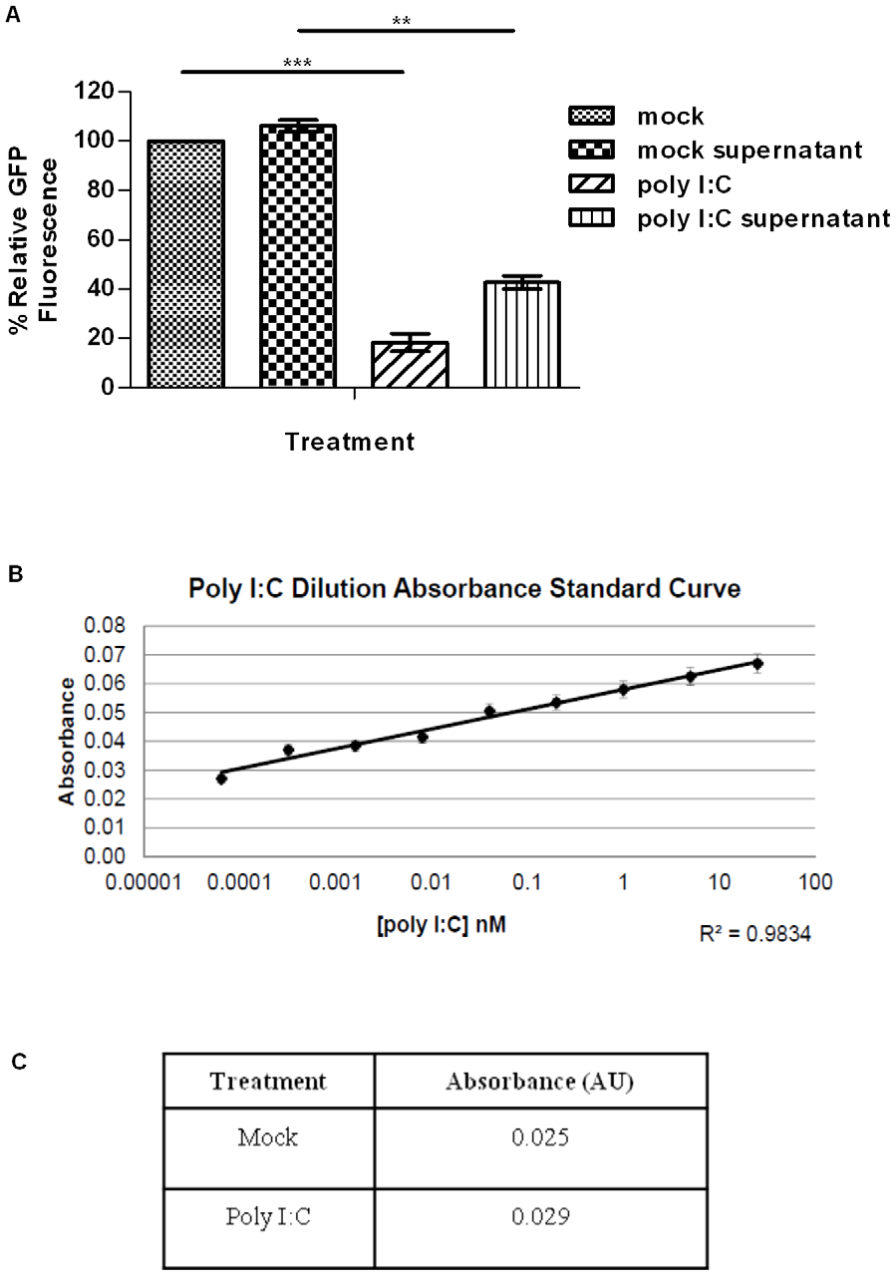
A soluble factor confers resistance to HSV-1 replication in IRF3^−/−^9^−/−^ MEFs. (A) MEFs were directly treated with 8.5 nM poly IC for 6 hours, or with supernatants from poly IC-treated cells, and subsequently challenged with HSV-1gfp. Initiation of HSV-1 replication, as measured by GFP fluorescence, is displayed relative to untreated (mock), infected cells, where fluorescence in each experiment was set to 100%. (B) A standard curve of poly I∶C in culture medium was generated. (C) The absorbance of supernatants collected after 6 hours of mock and poly I∶C treatment were collected and absorbance was compared to determine the amount of residual poly I∶C within supernatants of treated cells. *, p<0.05, **, p<0.01, ***, p<0.0001. Data are expressed as the mean ± SEM; n = 3.

We previously showed that IRF3^−/−^9^−/−^MEFs, which fail to make or respond to type I IFN, induce an antiviral response against HSV-1 and VSV following treatment with poly I∶C. The antiviral response against HSV-1 in these cells was found to occur earlier and was more potent than the response against VSV [16]. To determine whether this protection is conferred by a soluble factor, supernatants from poly I∶C-treated monolayers were transferred to naïve monolayers. The transferred supernatants were able to significantly limit initiation of HSV-1 replication on naïve monolayers, as indicated by the decrease in GFP fluorescence following challenge of monolayers with HSV-1gfp (Figure 1A). We routinely monitor GFP fluorescence as an indicator of initiation of virus replication [16]; in IRF3^−/−^9^−/−^MEFs, treatment with poly I∶C subsequently decreases HSV-1 infectious virus production by ∼15 fold (data not shown). To confirm that residual poly I∶C was not responsible for the protective effects, we measured the level of poly I∶C in the supernatants, relative to a standard curve of poly I∶C in medium (Figure 1B). The absorbance of poly I∶C-treated supernatants was only slightly higher than poly I∶C deficient (mock) supernatants (Figure 1C); moreover, poly I∶C concentrations within this low range do not confer resistance to HSV-1 infection in IRF3^−/−^9^−/−^MEFs [16]. Thus, the soluble factor present within the supernatants was not residual poly I∶C.

**Figure 2:**
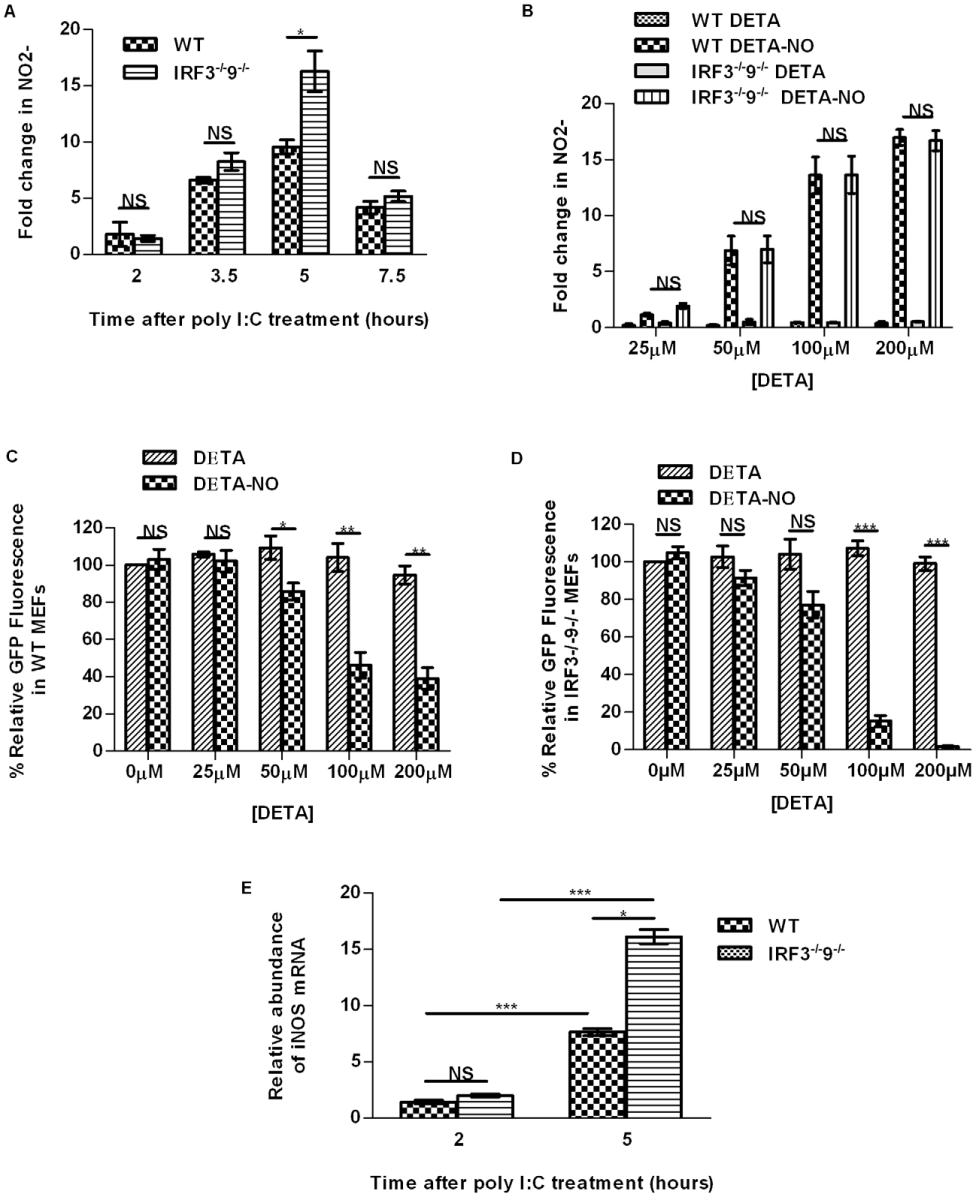
Nitric oxide is synthesized by iNOS in response to dsRNA in MEFs. (A) Nitric oxide was produced following treatment with poly I∶C in MEFs as determined by Griess assay. (B) DETA-NO, a nitric oxide donor, induced nitric oxide production in MEFs. Nitric oxide produced via DETA-NO limited initiation of replication of HSV-1gfp in WT (C) and IRF3^−/−^9^−/−^ (D) MEFs, as measured by GFP fluorescence, where fluorescence levels in mock treated cultures were set to 100%. DETA alone did not induce nitric oxide production and failed to reduce initiation of HSV-1 replication. (E) The relative abundance of iNOS mRNA, measured by qRT-PCR, in WT and IRF3^−/−^9^−/−^ MEFs treated with poly I∶C was found to be significantly higher in MEFs deficient for IRF3 and IRF9 after 5 hours of poly I∶C treatment in comparison to WT counterparts. Fold changes in transcript levels were compared relative to the housekeeping gene, *Gapdh*. *, p<0.05, **, p<0.01, ***, p<0.0001. Data are expressed as the mean ± SEM; n = 3.

As nitric oxide is a soluble factor and an important modulator of protection against DNA viruses such as HSV-1, we sought to determine if this molecule is involved in the antiviral response in the absence of IRF3 and IRF9. It was found via Griess assay, which measures the nitric oxide by-product nitrite, that these cells make nitric oxide within 2 hours of stimulation with poly I∶C. Nitric oxide production in both wild type (WT) and IRF3^−/−^9^−/−^MEFs peaked within 5 hours of treatment with poly I∶C (Figure 2A).

To confirm that nitric oxide production in IRF3^−/−^9^−/−^ MEFs decreased initiation of replication of HSV-1, varying concentrations of nitric oxide were added to untreated cells with the use of the nitric oxide donor, diethylenetriamineNONOate (DETA-NO). DETA-NO and the control compound diethylenetriamine (DETA) were added to cells at concentrations ranging from 0–200 µM. It was determined by Griess assay that DETA-NO used between 50–100 µM produced nitric oxide to levels similar to those observed in MEFs after treatment with 8.5 nM poly I∶C for 5 hours (Figure 2B). A concentration-dependent effect on initiation of HSV-1 replication in these cells was observed in both WT (Figure 2C) and IRF3^−/−^9^−/−^ (Figure 2D) MEFs. Between 50–200 µM DETA-NO was able to limit initiation of HSV-1 replication in WT and IRF3^−/−^9^−/−^ MEFs. At the higher concentrations, a greater effect of DETA-NO was observed in IRF3^−/−^9^−/−^MEFs. In each case, the control reagent DETA did not significantly contribute to nitric oxide production or affect initiation of HSV-1 replication.

### Induction of iNOS is important to the antiviral response in IRF3^−/−^9^−/−^ MEFs

Nitric oxide can be made by eNOS, nNOS, or iNOS depending on the stimulus and the cell type. Generally, synthesis has been shown to be a result of iNOS induction in response to viruses and viral components [29]. We used qRT-PCR to measure the levels of eNOS and iNOS in WT and IRF3^−/−^9^−/−^ MEFs. nNOS was not determined as this isoform is relatively restricted to neuronal cells [29]. While eNOS was not detected in either cell type, iNOS was basally detected in untreated cells to levels of approximately 0.3-fold in comparison to the housekeeping gene, *Gapdh* (data not shown). Transcript levels of iNOS increased in both WT and IRF3^−/−^9^−/−^MEFs upon treatment with poly I∶C within 2 hours (Figure 2E). Induction further increased after 5 hours of poly I∶C treatment, coincident with the timeframe in which protection against HSV-1 was observed. Levels of iNOS transcript were reduced to baseline measurements within 7.5 hours of poly I∶C treatment (data not shown).

**Figure 3:**
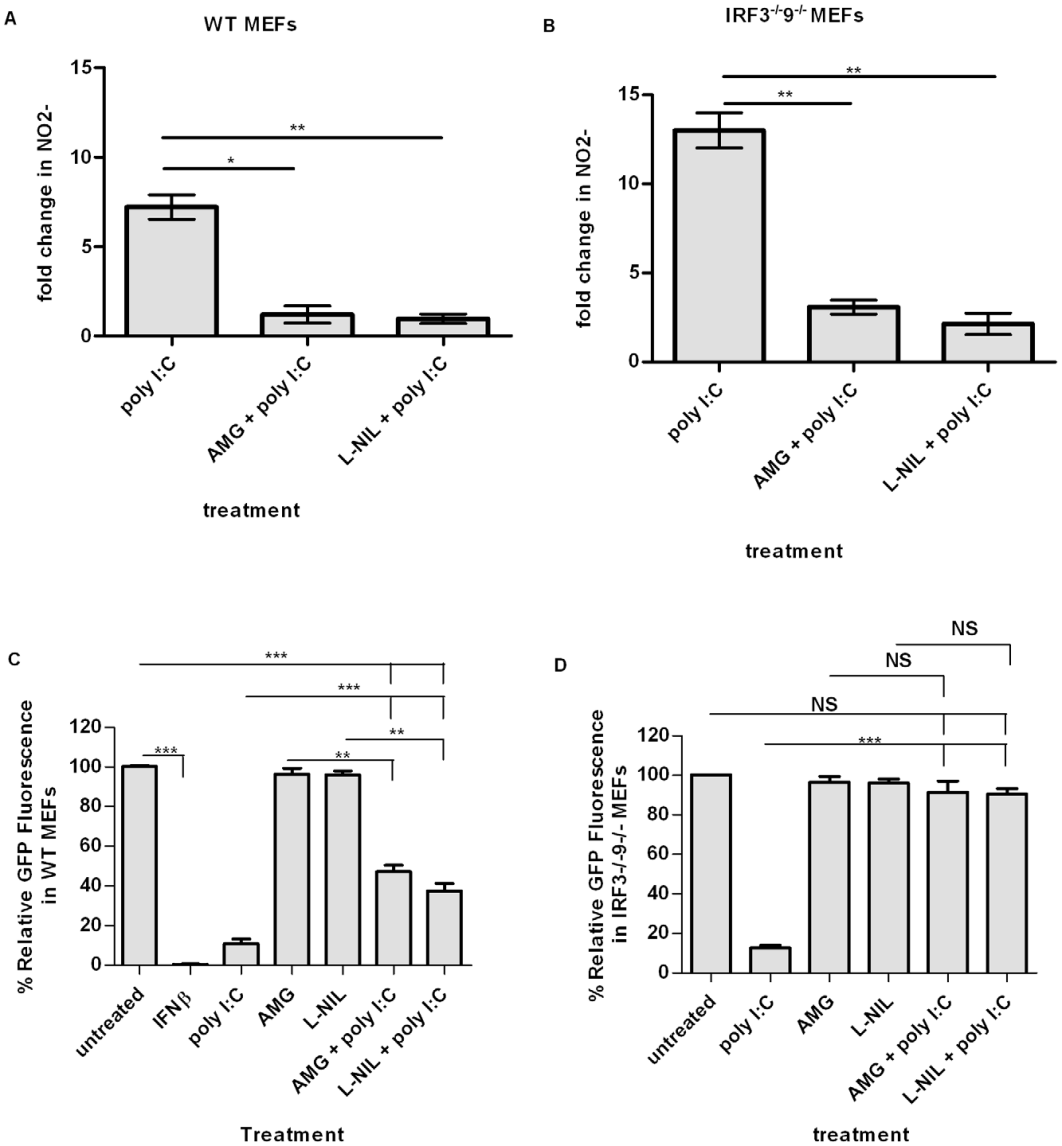
Nitric oxide made by iNOS is involved in the IRF3- and IFN-independent antiviral response. The efficacy of the iNOS inhibitors AMG and L-NIL to block nitric oxide production was determined by Griess assay following a 5 hour poly I∶C treatment of WT (A) and IRF3^−/−^9^−/−^ (B) MEFs. Initiation of HSV-1gfp replication in WT (C) and IRF3^−/−^9^−/−^ (D) MEFs following treatment with poly I∶C in the presence or absence of the iNOS inhibitors was measured relative to untreated cells, where fluorescence levels were set to 100% in each experiment. WT MEFs, which maintain the capacity to respond to IFN, are completely protected against HSV challenge following 24 hr pre-treatment with IFNβ. *, p<0.05, **, p<0.01, ***, p<0.0001. Data are expressed as the mean of three replicates ± SEM.

The classic iNOS inhibitor aminoguanidine hydrochloride (AMG) was used to determine the involvement of iNOS in the antiviral response observed against HSV-1 in IRF3^−/−^9^−/−^MEFs. Based on conflicting reports on the degree of isozyme selectivity of AMG [36], [37], N6-(1-iminoethyl)-L-lysine dihydrochloride (L-NIL), a compound more selective in iNOS inhibition [38], was also used. The efficacy of AMG and L-NIL as inhibitors of iNOS in poly I∶C-treated WT (Figure 3A) and IRF3^−/−^9^−/−^ (Figure 3B) MEFs was determined; there was a statistically significant reduction in nitric oxide production, as determined by Griess assay, upon treatment with poly I∶C in the presence of the inhibitors. Consistent with our hypothesis that dsRNA-mediated induction of nitric oxide limits initiation of HSV-1 replication, inhibition of iNOS with either AMG or L-NIL partially (Figure 3C) or fully (Figure 3D) restored initiation of HSV-1 replication in WT or IRF3^−/−^9^−/−^MEFs, respectively. The partial effect observed in WT MEFs is consistent with the ability of dsRNA to elicit IFN and ISG induction within these cells [16]. For reference purposes, the effect of IFNβ pre-treatment on limiting initiation of HSV-1 replication is shown (Figure 3C).

**Figure 4:**
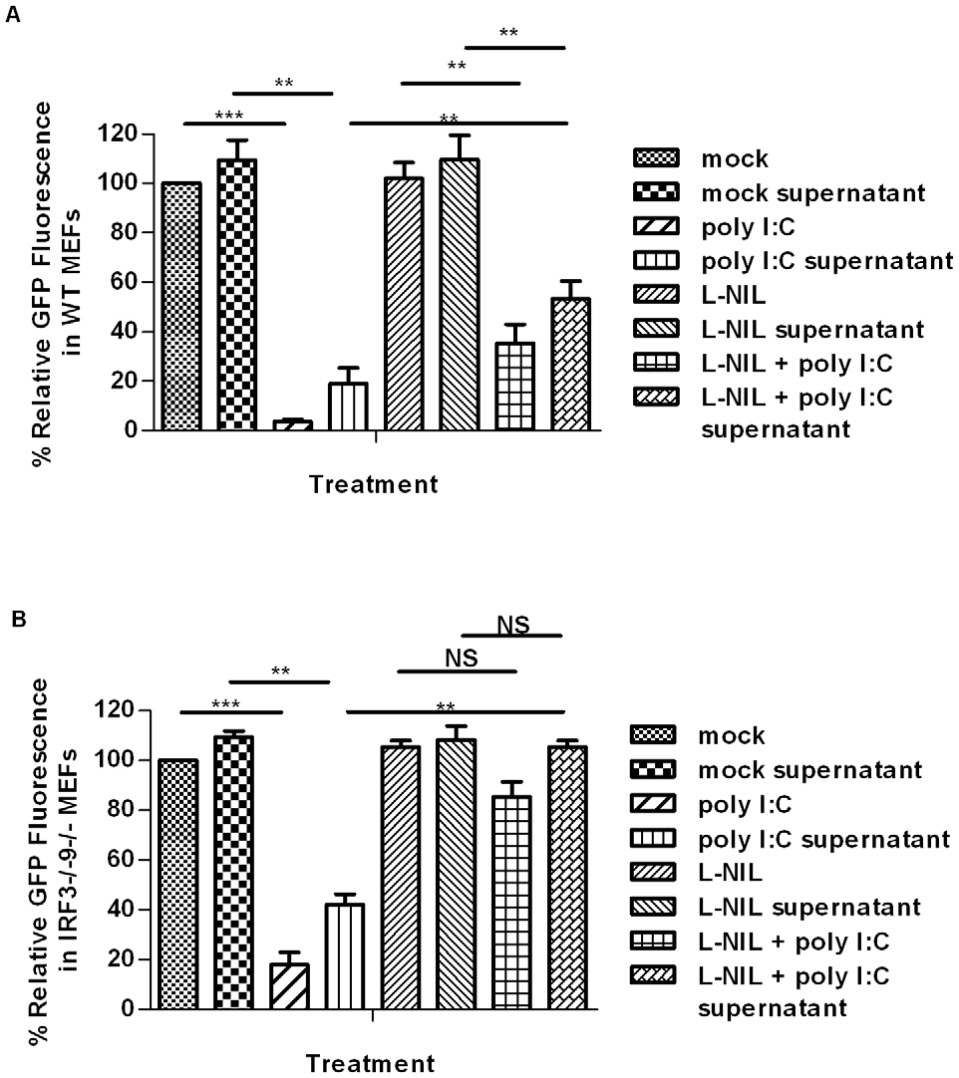
A component of the nitric oxide pathway provides antiviral protection in supernatants from dsRNA-treated MEFs. WT (A) or IRF3^−/−^9^−/−^ (B) MEFs were subjected to the indicated treatment, and initiation of HSV-1gfp replication was subsequently assessed 24 hours following virus challenge, as a measure of GFP fluorescence. *, p<0.05, **, p<0.01, ***, p<0.0001. Data are expressed as the mean of three replicates ± SEM, relative to mock treated samples, which were set at 100% in each experiment.

Data presented in Figures 1 and 2 show that MEFs treated with dsRNA produce nitric oxide and that a soluble factor found within supernatants can limit initiation of HSV-1 replication upon treatment of naïve cells. To our knowledge, there is no assay that directly measures nitric oxide within supernatants, *in vitro*. Thus, to determine whether nitric oxide or a component(s) of the nitric oxide pathway is responsible for protection of naïve monolayers following supernatant transfer, we transferred supernatants from cells treated with dsRNA in the presence or absence of the iNOS inhibitor L-NIL. We observed partial (Figure 4A) or complete (Figure 4B) restoration of initiation of HSV-1 replication following transfer of supernatants from cells treated with poly IC in the presence of L-NIL, relative to transfer of supernatants from cells treated with poly IC in the absence of an iNOS inhibitor. Consistent with the ability of WT MEFs to produce IFN and ISGs in response to dsRNA, only partial restoration of HSV-1 replication initiation was observed in these cells.

### NF-κB- and IRF1-mediated induction of iNOS contributes to protection against HSV-1 in the absence of IRF3 and IRF9

**Figure 5:**
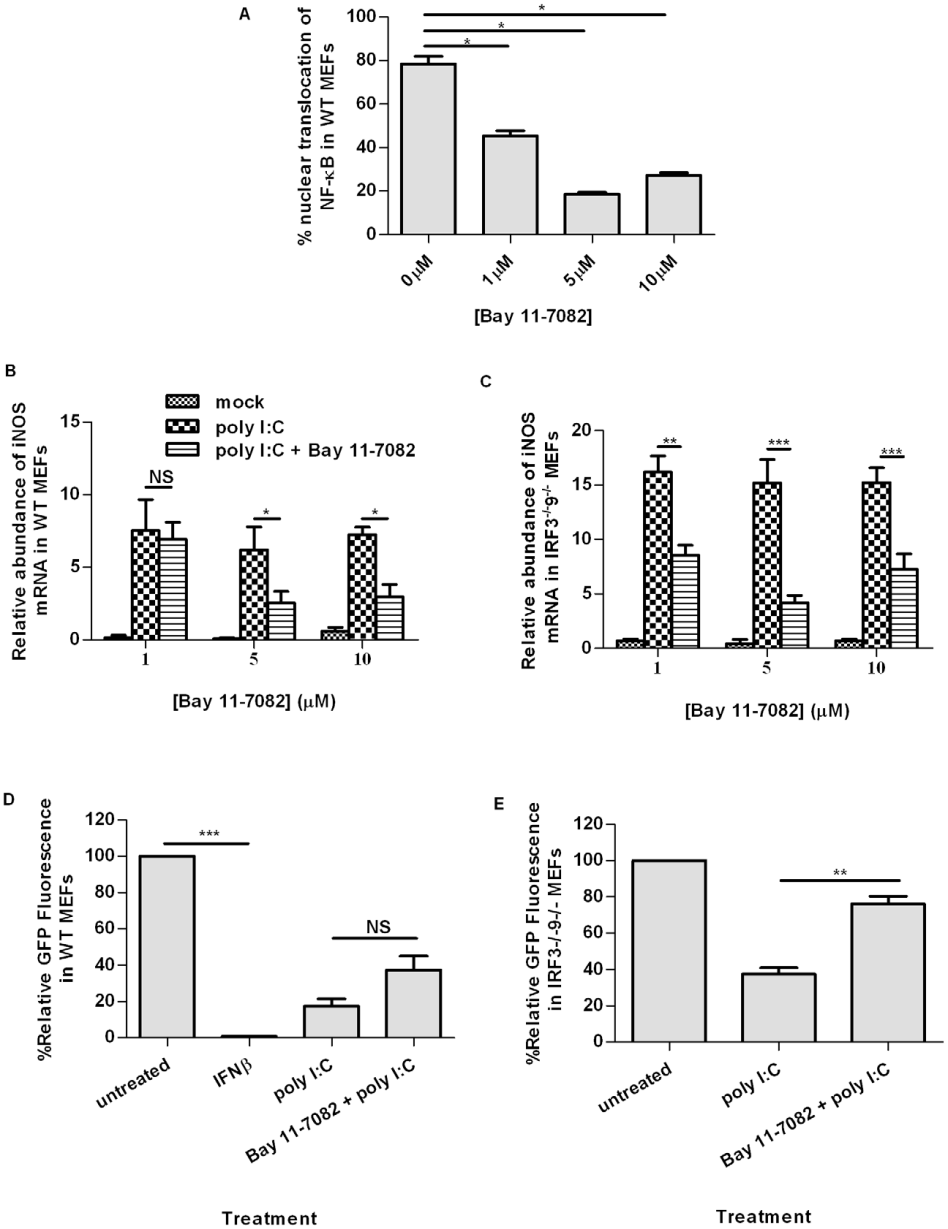
NF-κB contributes to iNOS induction and subsequent antiviral protection against HSV-1 in IRF3^−/−^9^−/−^ MEFs. (A) The effective concentration of Bay 11-7082 to inhibit NF-κB translocation to the nucleus after treatment with poly I∶C was determined. The effect of NF-κB inhibition by Bay 11-7082 on iNOS mRNA accumulation was measured by qRT-PCR in poly I∶C-treated WT (B) and IRF3^−/−^9^−/−^ (C) MEFs. Initiation of HSV-1gfp replication was quantified in WT (D) and IRF3^−/−^9^−/−^ (E) MEFs following a 5 hour poly I∶C treatment in the presence or absence of Bay 11-7082 and fluorescence compared to untreated monolayers, where fluorescence was set at 100% in each experiment. A 1-way ANOVA with a Tukey post-test was performed to compare efficacy of a range of NF-κB inhibitor concentrations. An unpaired t-test was performed to compare the antiviral response in cells that received poly I∶C treatment with and without Bay 11-7082; *, p<0.05, **, p<0.01, ***, p<0.0001. Data are expressed as the mean of three replicates ± SEM.

While the transcription factors NF-κB and IRF1 bind to the iNOS promoter to induce its transcription, these factors can signal independently of one another [39]. To confirm the role of NF-κB in the antiviral response observed in the absence of IRF3 and IFN, NF-κB was blocked using the inhibitor Bay 11-7082, which targets the phosphorylation of IκBα. Concentrations of Bay 11-7082 ranging from 0 µM–10 µM were tested and 5 µM was determined to be the optimal concentration to inhibit the nuclear translocation of NF-κB following treatment of MEFs with poly I∶C (Figure 5A). Inhibition of NF-κB significantly decreased the fold change in iNOS transcript expression as determined by qRT-PCR in WT and IRF3^−/−^9^−/−^ MEFs (Figure 5B, 5C). In an antiviral assay, inhibition of NF-κB with Bay 11-7082 resulted in increased initiation of HSV-1 replication in poly I∶C treated WT and IRF3^−/−^9^−/−^ MEFs (Figure 5D, 5E); the increase in initiation of virus replication was found to be significant in IRF3^−/−^9^−/−^ MEFs.

**Figure 6:**
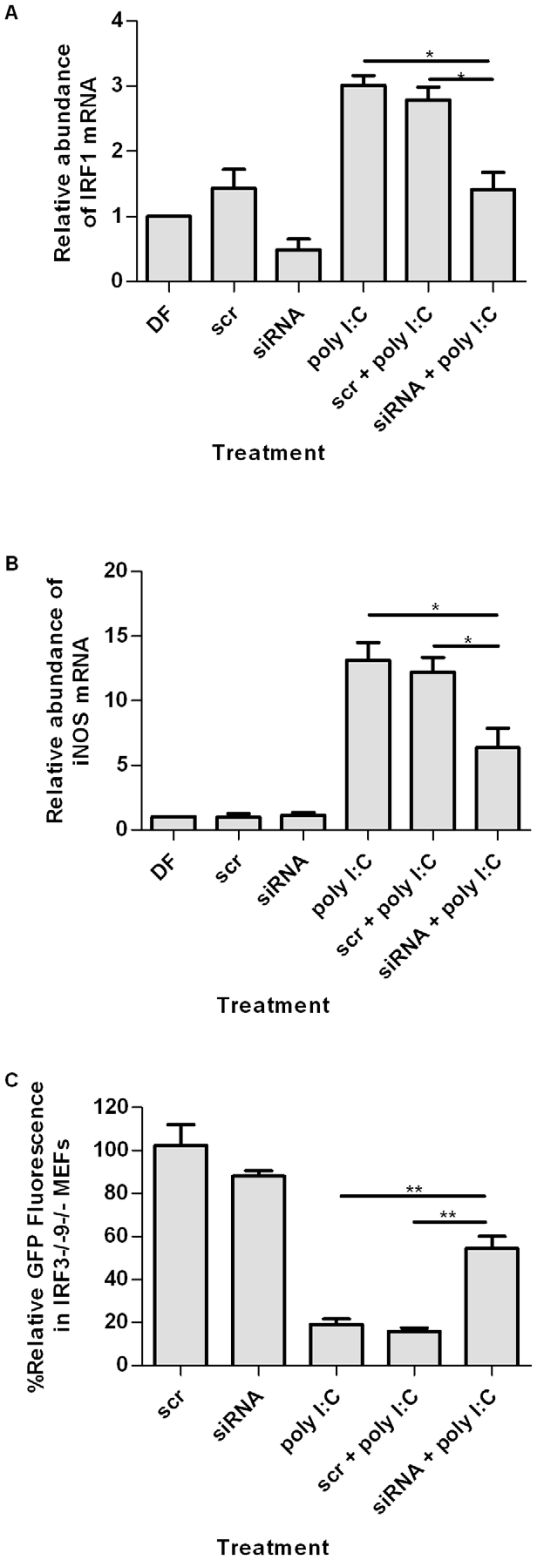
IRF1 contributes to iNOS induction in response to HSV-1 in MEFs. (A) Knockdown of IRF1 by siRNA in IRF3^−/−^9^−/−^ MEFs in the presence or absence of poly I∶C treatment. Fold change in iNOS mRNA accumulation (B) and initiation of HSV-1gfp replication (C) in IRF3^−/−^9^−/−^ MEFs treated with poly I∶C in the presence or absence of siRNA against IRF1. *, p<0.05, **, p<0.01, ***, p<0.0001. Data are expressed as the mean of three replicates ± SEM.

To assess the importance of IRF1 in iNOS induction and the antiviral response in the absence of IRF3 and IFN, an IRF1-targeting siRNA was generated. The control siRNA was a scrambled sequence of the IRF1 siRNA used to account for nonspecific effects. The efficacy of siRNA on IRF1 transcript levels was assessed by qRT-PCR (Figure 6A). IRF1 transcript was significantly reduced in IRF3^−/−^9^−/−^ MEFs treated with IRF1-specific siRNA compared with cells treated with transfection reagent DharmaFECT (DF) alone. Although treatment with poly I∶C increased levels of IRF1, these levels were significantly decreased upon addition of IRF1-targeting siRNA.

Quantitative RT-PCR analysis indicated that inhibition of IRF1 by siRNA significantly reduced iNOS transcript accumulation in comparison to control siRNA (Figure 6B). Furthermore, initiation of HSV-1 replication was significantly increased in poly I∶C treated IRF3^−/−^9^−/−^ MEFs in which IRF1 levels were decreased following siRNA treatment (Figure 6C). Taken together, these data suggest that both NF-κB and IRF1 contribute to iNOS induction and subsequent nitric oxide production in poly I∶C-treated IRF3^−/−^9^−/−^MEFs. Moreover, it is likely that these transcription factors function in additional pathways that contribute to antiviral activity, including production of type I IFN in WT MEFs.

## Discussion

Fibroblasts are well established as important effectors of the early innate immune response to pathogens [2]–[4] due to their rapid production of IFNβ and other cytokines [5]. Many pathogens, however, such as large DNA viruses, are particularly adept at subverting the type I IFN system, rendering their immediate environment IFN system defective. Thus, it is unclear how effector cells such as fibroblasts respond to specific pathogen triggers to protect themselves from a viral infection. DsRNA, a by-product of all viral infections, is a potent inducer of type I IFN, both as an intracellular and extracellular molecule [40]. Indeed, early recognition of DNA virus infection, including that of HSV-1, is mediated in part by dsRNA sensors [41], [42]. Although we previously found that fibroblasts can respond to dsRNA and protect themselves from subsequent virus infection in the absence of a type I IFN system, the mechanism of protection was unknown, as we failed to detect the production of any cytokines or chemokines [16].

Nitric oxide is an important cellular messenger involved in diverse physiological and pathological processes. Among its many properties, nitric oxide has potent antiviral and antibacterial activity. While macrophages are primary producers of nitric oxide in response to pathogens, nitric oxide production by dermal skin fibroblasts and rat embryonic fibroblasts has previously been shown to play a role in wound healing and host defense in response to bacterial PAMPs and inflammatory cytokines [43], [44]. With regards to the viral PAMP dsRNA, studies have shown induction of iNOS in human astroglia and bronchial epithelial cells [45], [46]. However, to our knowledge, there have been no reports of dsRNA eliciting a nitric oxide-mediated antiviral response in fibroblasts in the absence of type I IFN responses.

We set out to study the mechanism by which an antiviral response against HSV-1 occurred in the absence of IRF3, IFN and ISGs, particularly since this response was earlier and more robust than the cellular response against VSV, a small RNA virus that is exquisitely sensitive to the host IFN response [16]. We determined that the antiviral factor responsible for controlling HSV-1 replication was a soluble factor. Coincidentally, nitric oxide acts as a soluble antiviral factor that is more potent against DNA viruses in comparison to RNA viruses. We found that nitric oxide is rapidly produced by MEFs in response to the dsRNA mimetic poly I∶C, and serves to inhibit HSV-1 replication. It is unclear why the by-products of nitric oxide production appear to decline over time, as the Griess assay measures nitrite, one of two stable break down products of nitric oxide. However, recent studies show that both nitrate and nitrite can be recycled back into the nitric oxide pathway [47], which may explain this observation. The antiviral activity of poly I∶C-induced nitric oxide was confirmed by the addition of nitric oxide to MEFs using DETA-NO. While we cannot directly measure nitric oxide in cellular supernatants, we found that inhibition of nitric oxide production in MEFs reduced the antiviral activity within supernatants, suggesting that either nitric oxide, or a product of the nitric oxide pathway, serves as a soluble antiviral factor. Of interest, in WT fibroblasts, nitric oxide is a contributor, albeit minor, to the antiviral response; the ability to make and respond to type I IFN likely constitutes the major antiviral activity in these cells. However, in IRF3^−/−^9^−/−^ MEFs, the nitric oxide pathway appears to be the dominant antiviral pathway. These data suggest that nitric oxide is an important antiviral molecule in the absence of IFN, ISGs and other cytokines.

Previous studies have shown that HSV-1 is susceptible to the effects of nitric oxide *in vivo* in mice and rats [33]–[35]. Although nitric oxide is synthesized during the host response to pathogen invasion, its precise role remains unclear. Despite its antiviral activity, nitric oxide is not always beneficial, as it can promote the pathogenesis of HSV-1 by damaging cells in host tissues, thus aiding infection [29], [48]. It is unknown at this time whether the antiviral protection provided by nitric oxide *in vivo* is exerted in the form of cytotoxicity as a result of nitrative stress or by an alternative mechanism. *In vitro*, treatment of primary fibroblasts with dsRNA or DETA-NO does not elicit noticeable cytotoxic effects (data not shown), suggesting that the level of nitric oxide production that is sufficient to block virus replication is not linked to cytotoxicity. While it is well known that NO has antiviral activity, particularly against DNA viruses such as HSV and vaccinia virus [34], [49], [50], the mode of action remains to be fully elucidated. Thiol modification and protein nitrosylation are likely involved in the antiviral activity of NO [51]. Indeed, viral enzymes are an important target for NO [52], [53]. Although NO displays antiviral activity against several RNA viruses, including influenza virus [54], its capacity to induce DNA damage [55] likely explains its preferential targeting of DNA viruses.

We have found in this study the importance of iNOS as the enzyme by which nitric oxide is synthesized. This is not surprising, as iNOS induction in response to virus infection, as well as viral components, is well known [56]–[58]. During viral infection, nitric oxide production by iNOS is induced by cytokines such as IFN-γ; however, virus infection can up-regulate iNOS independently of such cytokines [29]. While MEFs can respond to IFN-γ, they do not make it in response to poly I∶C (data not shown), suggesting that in fibroblasts, iNOS is induced independent of IFN-γ.

The iNOS gene locus has low homology between human, rat and mouse sequences. As a result, the transcription factors involved in iNOS induction are species and cell type dependent [39], [59]. For example, regulation of nitric oxide production by iNOS in humans has been shown to be dependent on AP-1, but a binding site for this transcription factor is not present on the mouse iNOS promoter [59]. NF-κB and IRF1 are most commonly published as regulators of iNOS expression in various species [60]–[62]. Furthermore, virus infection leads to the activation of NF-κB and IRF1 [63], [64]. Here we have shown that regulation of iNOS expression is, at least in part, dependent on both NF-κB and IRF1. These data are consistent with other reports indicating that both transcription factors are important for iNOS expression in mice in response to dsRNA [39], [65]. Our data do not exclude, however, the involvement of other transcription factors or the likelihood that NF-κB and IRF1 function in contributing to the antiviral response in another capacity.

Overall, our data show that components of the nitric oxide pathway serve as alternative antiviral factors in the absence of the IRF3- and IFN-mediated signaling pathway, and that they also contribute to the antiviral response in WT cells. While we conclude that the nitric oxide pathway is important, we cannot rule out involvement of other pathways or factors. Furthermore, the factors involved in the antiviral response against VSV after 24 hours of poly I∶C pretreatment [16] are still unknown. As levels of nitric oxide began to decline within 7.5 hours of treatment with poly I∶C, it is unlikely that this pathway is involved in the antiviral response observed against VSV at this timepoint. These data emphasize the intricacies of the host response to different pathogens, and underscore the requirement of the host to have multiple strategies to counteract the immune evasion properties of viruses.

## Materials and Methods

### Ethics statement

Human ethics approval was not required as neither human subjects nor primary human tissues were utilized. Production of primary mouse embryonic fibroblasts was completed with full approval from the McMaster Animal Review Ethics Board. All work with viruses was completed with full approval from the McMaster Presidential Biosafety Advisory Committee.

### Cells and Viruses

Primary MEFs derived from WT C57Bl/6 and IRF3^−/−^IRF9^−/−^ mice [14] were maintained in alpha-minimal essential medium (MEM) supplemented with 15% fetal bovine serum (FBS), 100 U•ml^−1^ penicillin, 100 µg•ml^−1^ streptomycin and 2 mM L-glutamine. Experiments were performed with cells at passages four to eight. All cells were incubated at 37°C in a humidified 5% CO_2_ incubator as previously described [16].

Vero cells (ATCC) were maintained in Dulbecco's modified Eagle medium (DMEM) supplemented with 5% FBS. HSV-1 (KOS strain) expressing green fluorescent protein (HSV-1gfp) [66] was propagated on Vero cells. For viral infections, cells were split and seeded into dishes 24 hours prior to infection. Infections with HSV-1gfp utilized a multiplicity of infection of 0.1 particle forming units (PFU) per cell and occurred in serum-free alpha-MEM for 1 hour. This amount of virus is the maximal dose for which signal saturation in untreated cells does not occur. Following 1 hour of infection, the viral inoculum was removed and replaced with DMEM containing 1% methylcellulose. GFP fluorescence intensity was measured on a Typhoon Trio (GE Healthcare) 24 hours later and quantified using ImageQuant TL software.

### DsRNA and IFN treatments

Cells were mock-treated, or treated with 8.5 nM poly I∶C (GE Healthcare, Buckinghamshire, UK) in serum-free OptiMEM medium (Life Technologies) in the presence of 50 µg/ml DEAE-dextran (Pharmacia) for 1 hour. Full growth medium replaced OptiMEM for additional indicated amounts of time, unless otherwise noted. In all experiments, DEAE-dextran was used in controls not treated with poly I∶C to ensure that subsequent cellular responses were not influenced by the former. It was previously determined that 8.5 nM poly I∶C induces maximum protection in IRF3^−/−^9^−/−^ MEFs [16]; as such, this concentration was used to investigate the mediators of the observed antiviral response.

For IFN treatments, cells were pretreated with 100 U/ml IFNβ (provided by Dr. Brian Lichty, McMaster University) for 24 hours prior to challenge with HSVgfp.

### Supernatant transfer

Supernatants from mock-treated cells and cells treated with 8.5 nM poly I∶C for 5 hours were transferred to naïve IRF3^−/−^9^−/−^ MEFs and left on for another 5 hours. For experiments utilizing the iNOS inhibitor L-NIL, please refer to *Inhibition of iNOS* section, below. Cells were challenged with HSV-1gfp to determine if a soluble factor confers resistance to infection in the absence of both IRF3 and IRF9. Viral replication was quantified 24 hours post-infection as assessed by GFP fluorescence. To ensure no residual poly I∶C in the transferred supernatants was conferring resistance to HSV-1 replication, absorbance of the supernatants was measured. In complete MEF media, poly I∶C was serially diluted 1∶5 with concentrations ranging from 2.2×10^−5^–8.5 nM. This concentration range corresponds with that used in antiviral assays described previously [16]. A spectrophotometer was used to determine the absorbance of each concentration to derive a linear curve. To compare, absorbance of the supernatants at the time of transfer was also measured.

### Measurement of Nitric Oxide Production

MEFs were seeded in 96-well plates to approximately 70% confluency. After 24 hours, the cells were incubated with poly I∶C for 5 hours as described [16]. Following treatment with poly I∶C, the concentration of nitric oxide in the supernatants of MEF cultures was assessed by measurement of NO_2_^−^, an oxidized metabolite of nitric oxide. For this, a Griess reaction was performed as previously described [67]. Standards were prepared with known concentrations of NaNO_2_ (BDH) ranging from 0–20 µM prepared in alpha-MEM. Griess reagent (Sigma, USA) was prepared according to the manufacturer's instructions and subsequently added to standards and samples. The absorbance of the supernatants in the plates was read at 550 nm after a 10 minute incubation at room temperature with Griess reagent.

### Nitrite Release

WT and IRF3^−/−^9^−/−^ MEFs were seeded at a confluency of ∼80% overnight prior to treatments in 12-well plates for antiviral assays and in 96-well plates to assess nitrite release via Griess assay. The cells were treated with 0–200 µM DETA-NO (Sigma, USA) or with 0–200 µM of the control NONOate, DETA (diethylenetriamine; Sigma, USA) diluted in complete MEF media for 5 hours. Following treatment, cells were challenged with HSV-1 described earlier in an antiviral assay and viral replication was quantified as assessed by GFP fluorescence. Fresh DETA or DETA-NO was added after each medium change.

### Quantitative Real Time-PCR (qRT-PCR)

RNA was isolated from cells using TRIzol reagent (Life Technologies). A random 6-mer primer (0.2 ng) and 50 U of Superscript II (Life Technologies) were used to reverse transcribe 300 ng of DNase-treated RNA (DNA-free kit, Ambion, Austin, TX) in a total reaction volume of 20 µl. Subsequently, qRT-PCR was performed in triplicate using Universal PCR Master Mix and gene-specific TaqMan primers (Life Technologies) in a total volume of 25 µl.Data were analyzed via the ΔΔCt method. Gene expression was normalized to Gapdh, the housekeeping gene, and expressed as fold change over the mock-treated group (cells treated with DEAE-dextran alone). TaqMan specific primers used in this study include iNOS (Mm00440502_m1), IRF1 (Mm00515191_m1) and Gapdh (Mm99999915_g1).

### Inhibition of iNOS

The iNOS inhibitors AMG and L-NIL (Sigma, USA), were diluted in complete medium to a final in-well concentration of 10 µM. To investigate the efficacy of the iNOS inhibitors, cells were pretreated for 2 hours with iNOS inhibitors prior to a 5 hour treatment with poly I∶C. RNA was then collected using TRIzol and prepared for qRT-PCR as described above. Expression of iNOS transcript levels both with and without inhibitor were compared using TaqMan specific primers to determine efficacy of each of the iNOS inhibitors used.

To investigate the role of iNOS in the antiviral response, cells seeded the previous day in 12-well plates to 80% confluency were treated as described above. Cells were subsequently challenged with HSV-1gfp as indicated previously.

### NF-κB Inhibitor Preparation

The NF-κB inhibitor Bay 11-7082 (EMD, Gibbstown, NJ) was prepared in 1% DMSO, and used at concentrations ranging from 1–10 µM. Cells were incubated with this compound for 0.5 hours prior to mock and poly I∶C treatments. DMSO was included in mock- and poly I∶C-treatments.

### Immunofluorescence Microscopy

To determine the efficacy of Bay 11-7082 in inhibition of NF-κB in IRF3^−/−^9^−/−^ MEFs, cells were seeded on glass coverslips overnight at 60% confluency. Cells were either mock- or poly I∶C-treated with and without Bay 11-7082. Following 5 hours of treatment, cells were fixed with 10% formalin and subsequently permeabilized with 0.1% Triton X-100 diluted in 1× PBS for 10 minutes each. An overnight blocking step at 4°C followed in 1×PBS with 2% goat serum. A 1∶200 dilution of NF-κB p65 antibody (Santa Cruz Biotechnology, CA, USA) was added to the coverslips for 1 hour at room temperature. A secondary anti-rabbit IgG Alexafluor 488 antibody diluted 1∶1000 (Life Technologies) was hybridized for 1 hour at room temperature. Nuclei were stained with Hoescht dye diluted 1∶10000 for 10 minutes. All antibody and Hoescht dilutions were in 1×PBS with 2% goat serum. Images were taken and analyzed using a Leica DM IRE2 inverted microscope with Openlab software (Improvision). Nuclear translocation of cells that received poly I∶C and the inhibitor was plotted as a percent of untreated cells (mock-treated) corresponding to each dilution of Bay 11-7082.

### IRF1 siRNA

An oligonucleotide specific for IRF1, (5′-CAGACATCGAGGAAGTGAAGGATCA-3′) and a scrambled sequence (scr; 5′-CAGTAGCGAAGGAGTAAGGACATCA-3′) were designed (Thermo Scientific). The scrambled sequence was used to account for nonspecific knockdown of IRF1. The selected target sequences were tested so as not to match any known murine gene (other than murine IRF1) sequences by using NCBI nucleotide BLAST at http://www.ncbi.nlm.nih.gov/BLAST/. Transfection was performed according to the manufacturer's protocol. IRF1 siRNA and scrambled siRNA were used at a concentration of 50 µM. IRF1 gene expression following knockdown was quantified by qRT-PCR.

### Statistical Analyses

Data are represented as the mean of three replicates ± standard error of the mean (SEM). A one-way ANOVA with a Tukey post-test was used to compare the means of 4 concentrations of Bay 11-7082 in inhibition of NF-κB nuclear translocation. An unpaired t-test was used to compare the means of two groups where indicated. All analyses were performed using GraphPad Prism 5.0 software.
